# NMR-based metabolomics for investigating urinary profiles of metal carpentry workers exposed to welding fumes and volatile organic compounds

**DOI:** 10.3389/fpubh.2024.1386441

**Published:** 2024-08-07

**Authors:** Michele De Rosa, Ottavia Giampaoli, Fabio Sciubba, Federico Marini, Giovanna Tranfo, Renata Sisto, Alfredo Miccheli, Laura Tricarico, Anna Rita Fetoni, Mariangela Spagnoli

**Affiliations:** ^1^Department of Chemistry, Sapienza University of Rome, Rome, Italy; ^2^NMR-Based Metabolomics Laboratory (NMLab), Sapienza University of Rome, Rome, Italy; ^3^Department of Environmental Biology, Sapienza University of Rome, Rome, Italy; ^4^Department of Medicine, Epidemiology, Environmental and Occupational Hygiene, Istituto Nazionale Assicurazione contro gli Infortuni sul Lavoro (INAIL), Monte Porzio Catone, Italy; ^5^Catholic University of the Sacred Hearth, Faculty of Medicine and Surgery, Rome, Italy; ^6^Department of Neuroscience, Reproductive and Odontostomatological Sciences-Audiology Section, University of Naples Federico II, Naples, Italy

**Keywords:** NMR-based metabolomics, urinary profile, occupational exposure, welding fumes, volatile organic compounds, PLS-DA

## Abstract

**Introduction:**

Metal carpentry includes a wide range of work activities such as welding and cutting metallic components, use of solvents and paints. Therefore, the employees in these types of activities are mainly exposed to welding fumes and volatile organic solvents. Here, we present an NMR-based metabolomic approach for assessing urinary profiles of workers in the same company that are exposed to two different risk factors.

**Methods:**

The study enrolled 40 male subjects exposed to welding fumes, 13 male subjects exposed to volatile organic compounds of a metal carpentry company, and 24 healthy volunteers. All samples were collected, in the middle of the working week at fast. Thirty-five urinary metabolites belonging to different chemical classes such as amino acids, organic acids and amines were identified and quantified. Results were processed by multivariate statistical analysis for identifying significant metabolites for each working group examined, compared to controls.

**Results:**

Workers exposed to welding fumes displayed urinary increase in glutamine, tyrosine, taurine, creatine, methylguanidine and pseudouridine associated to oxidative impairment, while workers exposed to volatile organic compounds showed higher urinary levels of branched chain aminoacids.

**Conclusion:**

Our work identified specific urinary profile related to each occupational exposure, even if it is below the threshold limit values.

## 1 Introduction

Metal carpentry encompasses a wide range of work activities, such as assembling and disassembling of equipment, welding, and cutting metallic and electronic components as well as the use of solvents and paints.

Therefore, workers in these fields are exposed to different categories of chemicals: welding fumes (WF) and volatile organic compounds (VOC). Exposure to welding fumes is associated with various respiratory and cardiovascular diseases and since 2017 the IARC has classified them as confirmed carcinogens (group 1) (1). The processed electronic components that include different metals, such as Al, Sb, As, Be, Cd, Cr, Co, Hg, Ni, which can generate welding fumes composed of various toxic substances, as well as metals and the breathable fraction particles of oxidized metal (2–4).

Occupational exposure to VOCs has been classified in Groups 1 and 2 by IARC in 2010, and include substances such as benzene (Group 1), toluene (Group 3), xylene (Group 3), ethylbenzene (Group 2B), and styrene (Group 2A) (5).

The main health-adverse effects of occupational exposure to WF and VOC can induce an oxidatively generated damage of nucleic acids which in turn can lead to genotoxicity and inflammation, lung cancer and urinary bladder cancer (6–8).

Biological monitoring of exposure consists in the determination of dose biomarkers that measure the xenobiotic concentration by comparing it with the biological reference limits, of biomarkers of effect, which are used to evaluate the response of the organism to exposure in a subclinical state (9). Susceptibility indicators, on the other hand, express individual differences due to the metabolic phenotype. Human biomonitoring of dose and biochemical effect nowadays has great utility, providing an efficient and cost-effective means of measuring human exposure to chemical substances (10–12). Exposure to environmental toxins and human diseases lead to physiological changes that result in metabolite concentration variations (13–16). Metabolomics focuses on comprehensive characterization of small molecules (<1,000 Da), found in cells or organisms, in tissues or excreta, providing a snapshot of the metabolic dynamics in response to environmental exposure, pathophysiological stimuli and/or genetic modification (17). For that reason, metabolomics has demonstrated to be particularly useful in the identification of biomarkers, drug discovery and in studying organism-environment interactions at a molecular level (18).

In this study, we present for the first time an NMR-based metabolomic approach aimed at characterizing the urinary metabolic profiles of workers employed in the same metal carpentry company, according to their different work tasks.

## 2 Materials and methods

### 2.1 Experimental design

The study included 40 male workers exposed to welding fumes, 13 male workers exposed to VOCs, both ailing from the same company, and 24 male healthy volunteers (CTRL) (Table 1). The occupational exposure was verified as indicated by the occupational physician's report. Therefore, workers were subjected to health surveillance for urinary metal concentration, in case of welding exposure, and for urinary hippuric and methylhippuric acids, in case of xylene and toluene exposure.

**Table 1:** Characteristics of the enrolled subjects.

**Subjects**	**No**	**Age (range)**	**Smokers**	**Alcohol intake**
WE workers	40	53.4 (29–65)	9	0
VOC workers	13	44.9 (20–59)	7	0
CTRL	24	54.0 (18–70)	0	0

All samples were collected in the middle of the working week at the beginning of the work shift. All subjects gave their written informed consent to participate in the study. Occupational exposure to chemical agents was assessed by the employer and the workers involved in this study were equipped with the most appropriate personal protective devices, according to Italian legislation.

All experiments were conducted according to the Declaration of Helsinki and followed the International Code of Ethics for Occupational Health Professionals, published by the International Committee of Occupational Health (ICOH). The information gathered was used as aggregate data referring to the whole group of workers, with no risk of individual identification. This study was approved by the Ethical Committee of Fondazione Policlinico Universitario Agostino Gemelli, Università Cattolica del Sacro Cuore, protocol ID: 5117 (no-profit study), 03-08-2022. A written informed consent was obtained from all the involved subjects.

### 2.2 Sample preparation

Urine samples were collected by the workers in sterile plastic containers and then immediately transported refrigerated to the laboratory, where they were stored until analysis. One thousand two hundred microliter of urine were centrifuged at 11,000xg for 15 min at 4°C to remove the cellular debris. One hundred microliters of 3-trimethylsilyl-propionic-2,2,3,3-d4 acid (TSP) in D_2_O solution (2 mM, final concentration) were then added, as internal standard, to 1,000 μL of supernatant. The pH of urine was measured and adjusted at pH 7 by adding NaOH or HCl. An amount of 700 μL of each sample were then transferred to cryovials and stored at −80°C. Finally, the samples were transferred to precision NMR tubes and subjected to NMR analysis.

### 2.3 NMR acquisition and processing parameters

All samples were acquired by the JEOL ECZR-JNM spectrometer, equipped with a magnet operating at 14.1 Tesla and 600 MHz for proton resonance and with a cryogenic probe. The detailed parameters of acquisition have been described in previous studies (19). Subsequently, monodimensional ^1^H-NMR spectra were processed using ACD Labs software v.12.0 (Advanced Chemistry Development, Inc., 8 King Street East, Toronto, ON), and then we multiplied the free induction decays (FID) with an exponential function LB = 0.3 Hz, applied the Fourier Transform. All the spectra were manually phased and baseline corrected, by applying the baseline correction FID reconstruction (BCFR) procedure, as also reported elsewhere (20).

Bidimensional experiments have been also carried out on selected samples in order to univocally assign each resonance to the metabolites. Total correlation spectroscopy (TOCSY) ^1^H-^1^H experiments, heteronuclear single quantum coherence (HSQC) ^1^H-^13^C experiments have also been carried out according to Buonaurio et al. (21).

The assignment of the resonances was performed by the analysis of cross-correlated signals in 2D spectra and by comparison with the literature and open access databases (22). Only the molecules unequivocally identified were considered for the study, and their quantification was performed by integration of their NMR signals. The selected resonances were manually integrated and then normalized for the number of protons generating the signal. These values were compared with the normalized integral of TSP (internal concentration standard) and the obtained concentrations were further normalized for creatinine concentration, referred to the singlet signal at 4.05 ppm, free from overlaps with other signals. Quantities were finally expressed as μmol/mmol of creatinine.

### 2.4 Statistics for metabolomic analysis

Principal component analysis has been applied on data expressed as μmol/mmol of creatinine, after autoscaling, in order to highlight spontaneous grouping or the presence of any outliers.

Subsequently, supervised Partial Least Square discriminant analysis (PLS-DA) has been carried out to build a multi-parameter regression model and to identify variables significant for discriminating between the classes. Furthermore, for the validation process, a double-cross validation (DCV) have been applied according to previous studies (23, 24) and the performances of the method were summarized by the following figures of merit: sensitivity, specificity, accuracy and average correct classification rate.

The choice of significant variables has been done according to their weights on canonical variates (CVs) and the Variables Important in Projection (VIP) criterion (25). Variables with high weight and VIP major than 1 were chosen. For the univariate statistical analysis primarily Shapiro-Wilk (26) and Brown-Forscythe tests have been applied in order to evaluate the normality and the homoskedasticity of the distribution for each variable. Then, according to test results, Wilcoxon rank sum test or Student's *t*-test have been applied. Statistics was carried out employing MatLab 2023a (27) (the MathWorks, Natick, MA) and in-house written functions.

## 3 Results

**Figure 1:**
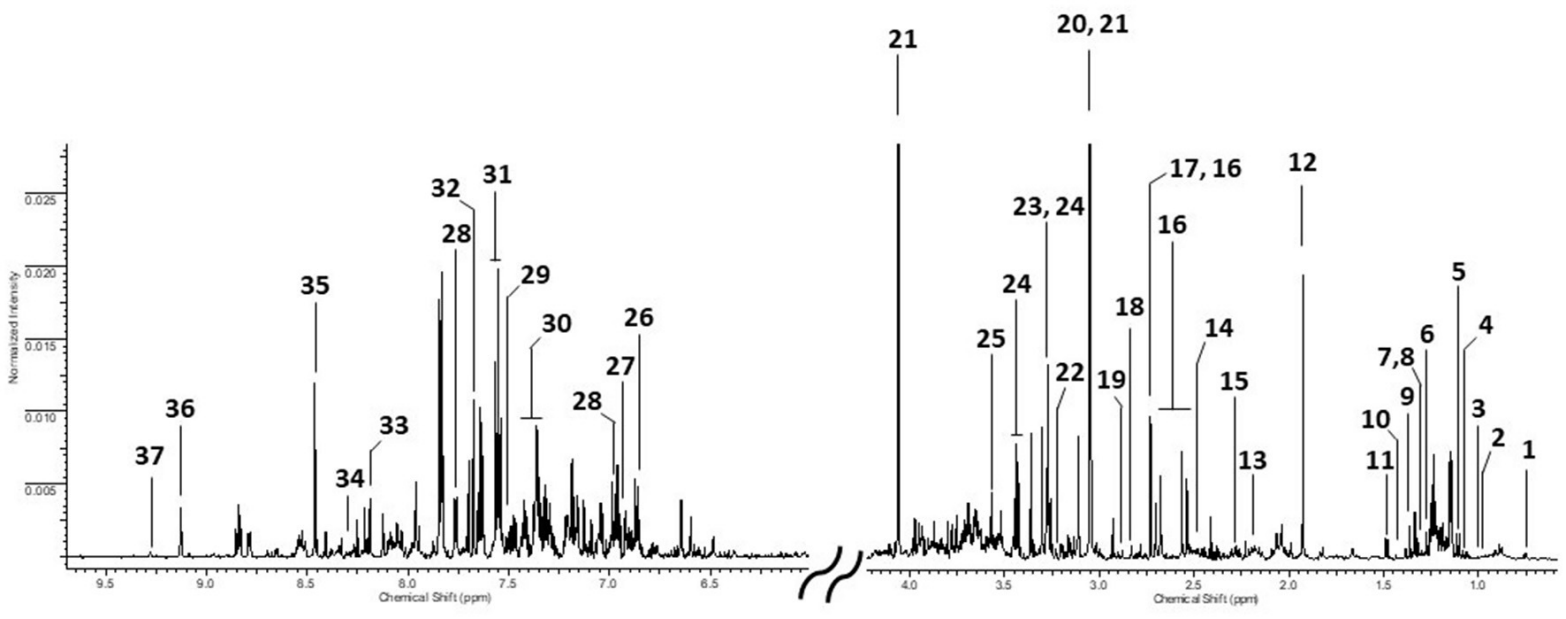
Urinary ^1^H NMR spectrum from a WE worker. 1: U01; 2: Valine (Val); 3: Isoleucine (Ile); 4: 3-hydroxyisobutyrate (3-HIBA); 5: Erythro-2,3-dihydroxybutyrate(Erythro-2,3-DHB); 6: 3-Hydroxy-3-methylbutyrate (3-H-3-MB); 7: Lactic acid (LA); 8: Threonine (Thr); 9: 2-Hydroxyisobutyrate (2-HIBA); 10: Dimethylmalonic acid (DMMA); 11: Alanine (Ala); 12: Acetic acid (AA); 13: N-acetylglutamine (NAcGln); 14: Glutamine (Gln); 15: p-Cresol sulfate (p-CrS); 16: Citric acid (CA); 17: Dimethylamine (DMA); 18: Methylguanidine (MG); 19: Trimethylamine (TMA); 20: Creatine (Crt); 21: Creatinine (Crtn); 22: Choline (Chn); 23: Taurine (Tau); 24: Trimethylamine-N-Oxide (TMAO); 25: Glycine (Gly); 26: 4-Hydroxyphenylacetic acid (4-HPAA); 27: Tyrosine (Tyr); 28: 4-Hydroxybenzoic acid (4-HBzA); 29: Tryptophan (Trp); 30: Phenylacetylglycine (PAG); 31: Hippuric acid (HippA); 32: Pseudouridine (PSI); 33: Hypoxanthine (Hyp); 34: N1-Methyl-2-pyridone-5-carboxamide (2PY); 35: Formic acid (FA); 36: Trigonelline (Trig): 37: 1-Methylnicotinamide (1-MNA).

An assigned urinary spectrum is reported in Figure 1. Comparing the different sample categories, no qualitative differences were found, but only quantitative ones. Therefore, we reported the spectrum of a welding fume exposed worker as an example. Thirty-five urinary metabolites, belonging to different chemical classes from aminoacids to organic acids and amines as well as molecules involved in purines (Hypoxanthine) and pyrimidines (Pseudouridine) pathways, have been identified and quantified. Whereas the unsupervised PCA did not show any spontaneous grouping between classes, nor even less the presence of outliers to exclude in further analysis, we have only reported here results obtained from supervised PLS-DA. However, for sake of completeness, we added this information in Supplementary Figures S1, S2.

### 3.1 Urinary profile of welding fumes exposed workers

**Figure 2:**
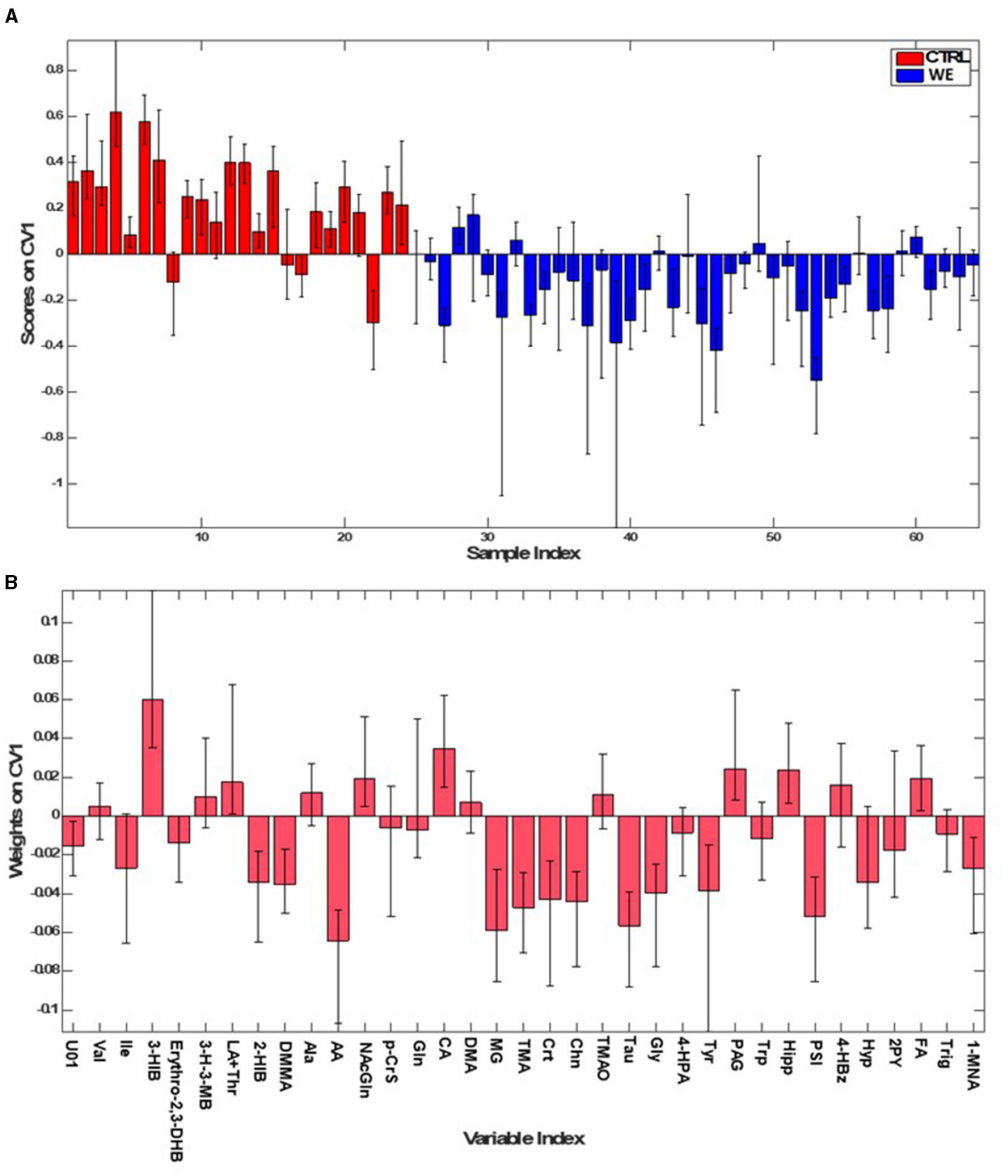
PLS-DA model for classifying WE (blue) and CTRL (red). **(A)** Sample scores; **(B)** variables weights along the only direction of maximum discrimination (first canonical variate) of the model.

The first PLS-DA model (Figure 2) was built to identify differences in urinary metabolic profile between the group of healthy volunteers (CTRL) and the workers in the welding sector (WE). The model allowed for the prediction of participant class belonging with 84.2 ± 2.9% accuracy, corresponding to an average correct classification rate of 83.4 ± 2.9%. The model showed sensitivity and specificity values for the WE group as compared to CTRL of 86.6 ± 3.6% and 80.3 ± 4.2%, respectively.

The contribution of individual metabolites to the discrimination can be appreciated by inspecting the sample scores along the only canonical variate (CV) (i.e., direction of maximum discrimination) of the model and the corresponding variables weights defining the projection (Figure 2).

**Figure 3:**
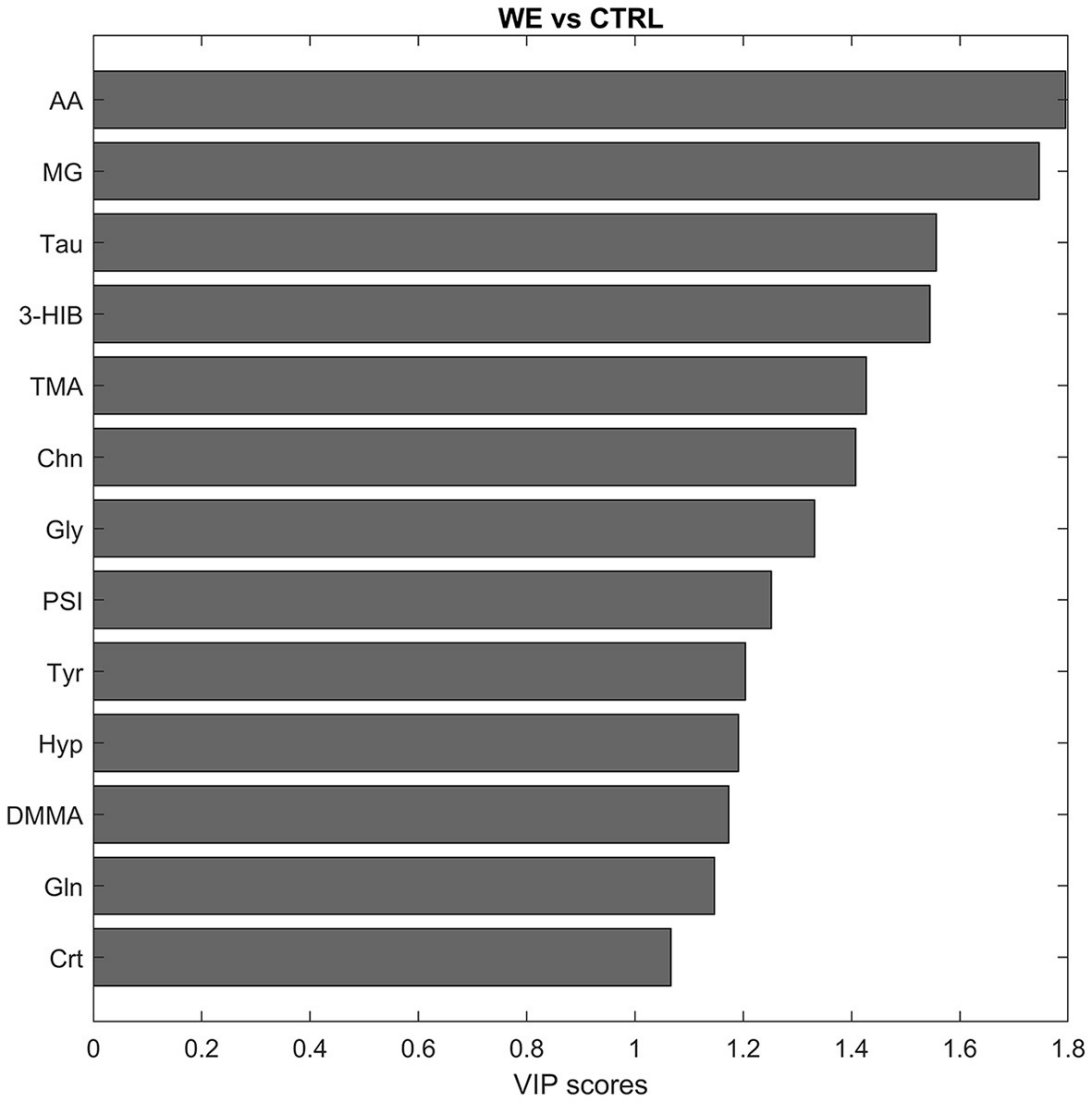
Discriminant metabolites after PLS-DA between CTRL and welding fumes exposed workers (WE) in descending order of VIP score.

In addition, for the selection of variables deemed interesting for the model, also the VIP scoring profile (Figure 3) was taken into account, ultimately considering as significant eleven variables involved in the characterization of the urinary profile of workers. In particular acetate (AA), methylguanidine (MG), Taurine (Tau), Trimethylamine (TMA), Choline (Chn), Glycine (Gly), Pseudouridine (PSI), Tyrosine (Tyr), dimethylmalonic acid (DMMA), creatine (Crt). The only variable significant for the CTRL group was 3-hydroxyisobutirate (3-HIB).

**Figure 4:**
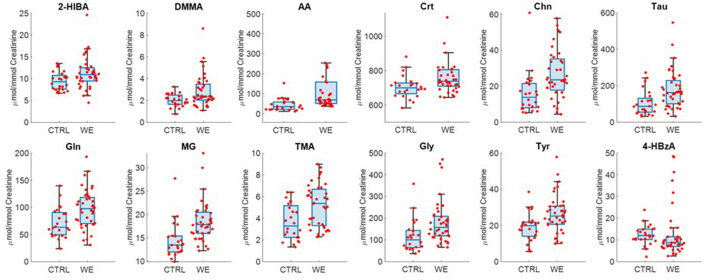
Selected metabolites discriminating controls (CTRL) and welding fumes exposed workers (WE).

At the same time, metabolites characterizing each type of exposure were observed by univariate statistical analysis (Figure 4). In fact, the urinary profile of welders is characterized by higher levels of 2-hydroxyisobutyrate (2-HIB), DMMA, AA, Crt, Chn, Tau, glutamine (Gln), MG, TMA, Gly, Tyr.

### 3.2 Urinary profile related to volatile organic compounds exposure

Secondly, in order to evaluate any effects on the metabolic profile of workers exposed to VOC, we performed a further PLS-DA, comparing VOC with the same control group.

The model did not show an excellent discrimination between two groups considered. In particular it showed an accuracy of 77.0 ± 5.3%, with sensitivity and specificity of 76.3 ± 9.0% and 77.3 ± 5.1% respectively in correctly classifying VOC from CTRL. As shown in Supplementary Figure S3, scores on CV1 present great error bars and some of the samples were misclassified.

**Figure 5:**
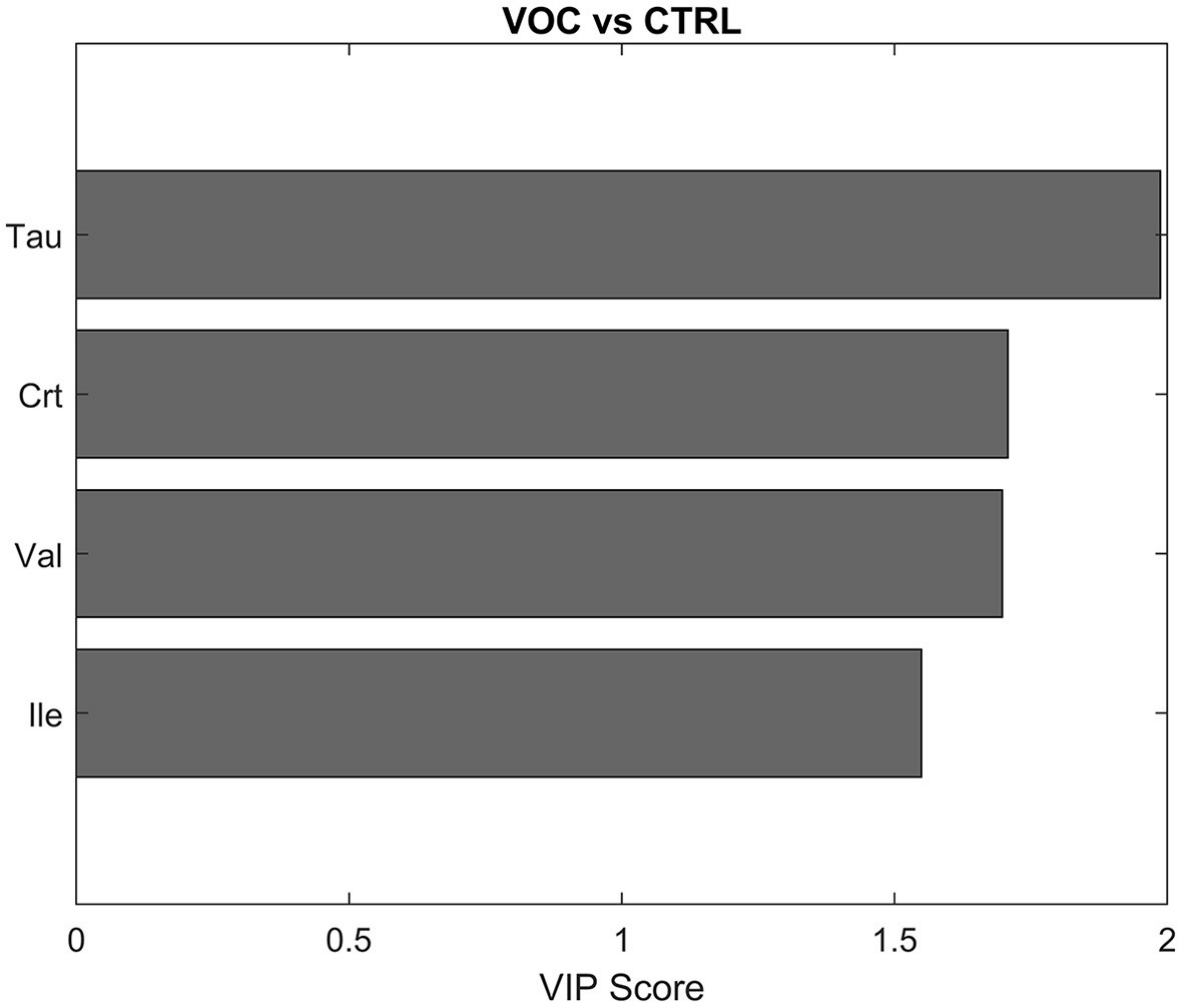
Discriminant metabolites after PLS-DA between controls (CTRL) and volatile organic compounds exposed workers (VOCs) in descending order of VIP score.

For that reason, the model was rebuilt with a subset of variables that showed the highest VIP values (Supplementary Figure S3) and hence the highest discriminant power for the model, in particular Tau, Crt, Val, Ile (VIP > 1.5), which contributed to VOC classification, as shown in Figure 5.

**Figure 6:**
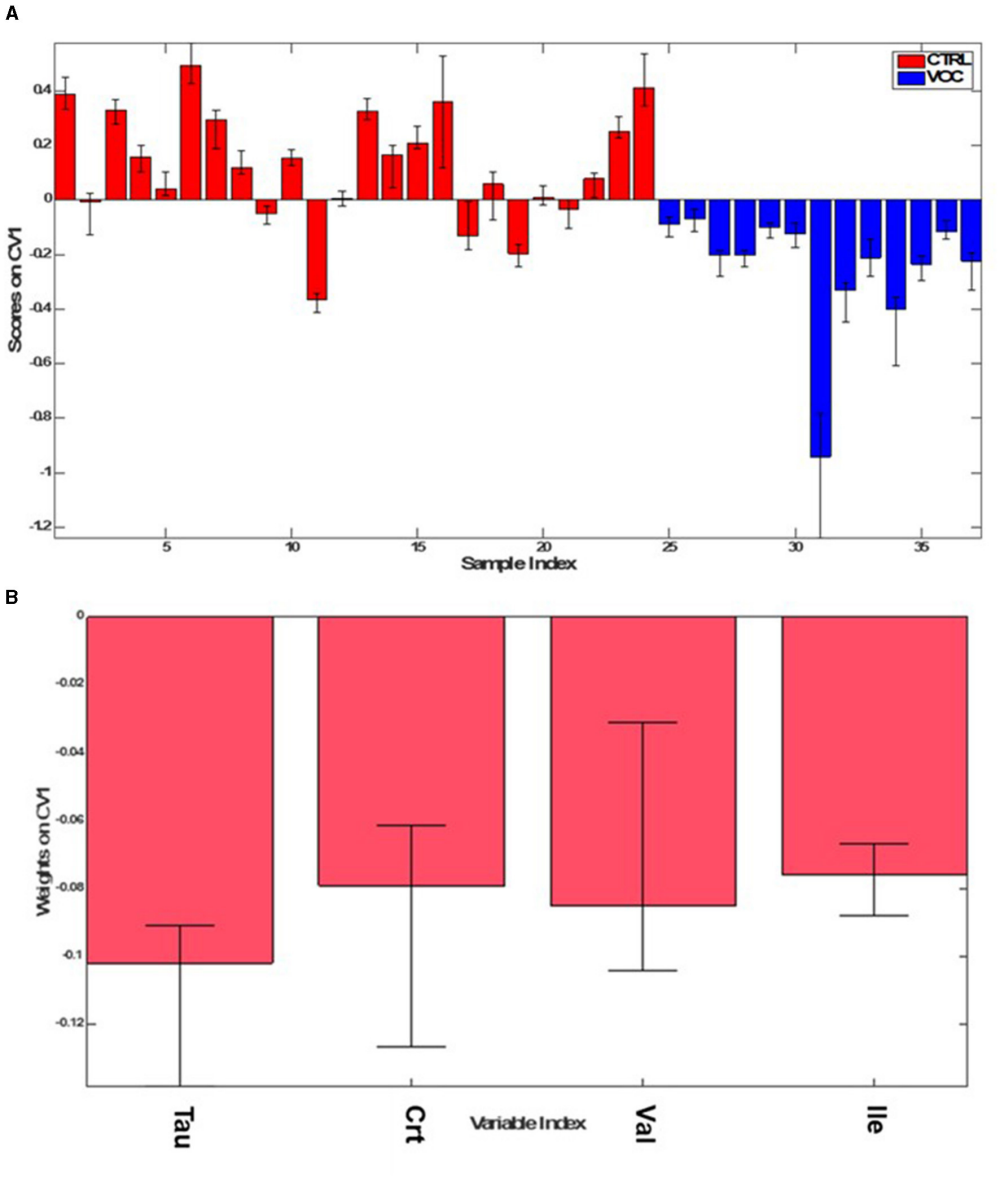
PLS-DA scores **(A)**, weights on CV1 **(B)** plots for the comparison between the CTRL (red) and VOC workers (blue).

The new model (Figure 6) indeed showed higher overall accuracy (89.6 ± 2.1%) and an average correct classification rate of 91.6 ± 2.1%, with sensitivity and specificity values of 98.3 ± 3.2% and 84.9 ± 3.0% respectively, for correctly classifying VOC from CTRL group.

**Figure 7:**
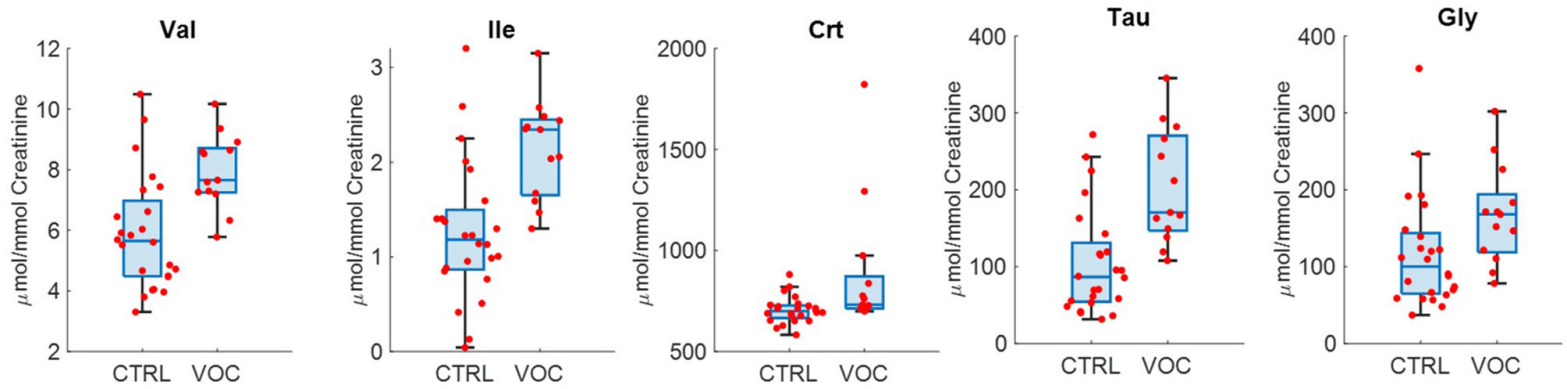
Selected metabolites discriminating controls (CTRL) and volatile organic compounds exposed workers (VOC).

The urinary profile of those exposed to VOC is characterized by higher levels of Val, Ile, Crt, Tau, Gly (Figure 7) as emerges from the univariate analysis.

Furthermore, metabolites which had a common trend for workers exposed both to welding fumes and those exposed to VOCs were, Tau, Crt and Gly, being discriminant for both models and in univariate analysis.

## 4 Discussions

As stated in the previous section, from the results obtained, a specific urinary profile of each exposure was identified, and intriguing findings have been found. In particular, higher urinary concentrations of Gln, Tyr, MG, PSI, TMA, 2-HIB, AA were found significantly higher in welding exposure.

Glutamine is generated by glutamine synthetase (GS) which catalyzes the condensation of glutamate and ammonia. In fact, ammonia is continuously produced and consumed throughout the human body during the metabolism of amino acids, purine and pyrimidine derivatives, polyamines. Ammonia is a neurotoxic compound and is detoxified by Gln synthetase which catalyzes the condensation of ammonia with glutamate (Glu) (28). As mentioned earlier, exposure to welding fumes include not only metals or metals oxides but also high emission rate of NOx, with NO being the most abundant (29, 30). It was demonstrated in Wistar rats that inhaled NO undergoes a series of biotransformations, which include absorption and conversion in the blood to ${\text{NO}}_{2}^{-}$/${\text{NO}}_{3}^{-}$, and subsequently the reduction of these intermediates to NH_3_ by the gut microbiota (31–33). Higher levels of glutamine for welding fumes exposed workers could hence indicate an increased activity of glutamine synthetase enzyme for the detoxification of ammonium ions deriving from inhaled NO.

Tyrosine is synthesized by phenylalanine hydroxylase, which catalyzes the hydroxylation of essential amino acid phenylalanine, a reaction that occurs mainly in the liver (34).

Increased concentration of urinary amino acid L-Tyrosine was found after exposure to Cd, also in case of low-level of exposure and was mostly associated with alteration to the oxidative state (35).

Methylguanidine is synthesized by nucleophilic attack on creatinine by direct molecular oxygen (36, 37) in hepatic peroxisomes. Since the function of peroxisomes is the scavenging of reactive oxygen species (ROS) (38), an increase of MG could confirm the hypothesis of an altered oxidative state in WE workers.

Pseudouridine (PSI) is a post-transcriptional RNA modification and is the most abundant modified nucleoside in RNA. Its urinary levels reflect RNA turnover and, indirectly, protein turnover. Its higher urinary levels could be associated with higher RNA turnover and hence a higher protein turnover (39), indirectly reflecting higher impairment of oxidative state.

TMA is a metabolite produced by the gut microbiota and which derives mainly from choline and carnitine (40). 2-HIBA is a short chain organic acid which had been previously associated with the presence of *Faecalibacterium prausnitzii*, frequently involved in dysbiosis (41, 42). Their significant increase in workers exposed to welding fumes could be associated with a different composition of the intestinal microflora, considering that such alteration due to occupational exposure had already been observed in several studies (43–45).

Acetate is also a product of the gut microbiota; however in a recent work, it has been showed a plausible reaction pathway for the generation of acetate by nucleophilic attack on pyruvate by the ROS generated from hydrogen peroxide (46).

On the other hand, VOC exposed workers displayed higher urinary levels of Valine (Val), Isoleucine (Ile), suggesting alterations in metabolism of branched chain amino acids (BCAAs). BCAAs pathway was found altered in a group of healthy workers exposed to benzene (47).

The two groups of workers present common trends of the same metabolites, defined by higher levels of Gly, Tau, Crt therefore not associated with the particular type of exposure. A key role could be played by ROS, whose increase is notoriously associated with both types of exposure taken into consideration in our study (48). In fact, regarding VOCs, several compounds have been demonstrated to possess cytotoxic effects linked to the activation of cell death processes mediated by an increase in ROS (49). Regarding welding fumes, the nature of the chemical composition is different based on the material, the process and the shielding gas used. In addition, it is known that during the welding process there is formation of nitrogen oxides and ozone due to UV dissociation of molecular oxygen (50); and inhalation exposure to these substances has once again been shown to cause an increase in the concentration of reactive oxygen species at the mitochondrial level, mediators of cellular oxidative damage (51).

Glycine (Gly) is a non-essential amino acid that the organism is capable of synthesizing starting from serine; it plays important roles at a physiological level including a plastic function in protein synthesis. Indeed, in association with hydroxyproline, Gly is capable of generating the peculiar helical structure of collagen, main constituent of the extracellular matrix (52). Besides immunoregulatory properties (53), several studies have demonstrated the ability of glycine to act as an antioxidant by inhibiting the production of ROS in human neutrophils (54). Furthermore, Gly is involved, with Cys and Glu, in the glutathione (GHS) synthesis, a natural tripeptide with high antioxidant properties, capable of preventing the oxidatively generated damage through the removal of ROS (55).

Taurine in physiological concentrations also has significant potential scavenging peroxyl radicals, nitric oxide, and superoxide donors (56). Therefore, the increase in urinary levels of these species could be the result of a response of the organism against the alteration of the oxidative state.

Creatine is a nitrogenous organic compound synthesized from L-Arginine and Gly, in two successive phases which involve the formation of precursors such as guanidinoacetate and s-adenosyl-l-methionine (57). The enzyme creatine-kinase catalyzes the reversible phosphorylation of the guanidino group of creatine to phosphocreatine involving an ATP molecule as a donor of a phosphate group. Creatine kinases are enzymes sensitive to oxidatively generated damage and therefore to the increase in ROS levels (58–60), and higher levels of creatine observed in workers urine could be related to the increase of these species.

All these species seem to suggest the triggering of a non-specific response mechanism of the organism following both exposures and higher levels of Gly, Tau, Crt could indicate a urinary pattern probably associated with alteration of ROS balance.

## 5 Conclusions

In this study, for the first time, the biochemical effects of exposure to welding fumes and volatile organic compounds were observed in the urinary metabolic profile of metal carpentry workers. The findings allowed us to identify metabolite alterations related to each exposure, even though the complexity of the occupational environment, with the workers having different tasks in adjacent non compartmentalized spaces.

Firstly, workers exposed to welding fumes could have higher oxidative stress as the urinary increase in Gln, Tyr, Gly, Tau, Crt, MG, PSI, AA could be traced back to an increase in reactive oxygen species, largely formed by metal welding operations, and these data agree with what has already been observed in the literature. An involvement of gut microbiota has also been hypothesized, since microbiota metabolites 2-HIB, AA, TMA, Chn and were significantly higher in workers. Secondly, a characteristic profile of those exposed to VOCs was also observed, based on higher urinary levels of BCAAs.

Thirdly, workers employed in the same company with different work tasks, regardless the exposure, present common alteration in urinary levels of Gly, Tau, Crt.

This study is surely limited by the small number of subjects, but this limitation is strictly related to the principle of voluntary participation and to the production structure, as Italy is dominated by small and medium size enterprises. Furthermore, it is important to consider that metabolic profiles could be influenced by different external factor including diet and lifestyle, even if we tried to choose groups as homogeneous as possible in terms of sex, age, smoking and alcohol intake. Considering these observations, it could be interesting to consider a follow-up of this study enrolling an increased number of subjects, in particular for VOCs exposed workers and healthy volunteers' groups, at the aim of validating the identified possible biomarkers specific to each occupational exposure. In addition, the future possibility of combining the metabolic urinary profile of workers with other biological parameters, such as dose biomarkers (exposure) and others putative effect biomarkers including those of oxidative stress, from a multiplatform analysis perspective could surely enrich the described methodology for the assessment of possible health effects related to the exposure, even if it is below the threshold limit values.

## Data availability statement

The original contributions presented in the study are included in the article/Supplementary material, further inquiries can be directed to the corresponding author.

## Ethics statement

The studies involving humans were approved by Ethical Committee of Fondazione Policlinico Universitario Agostino Gemelli, Università Cattolica del Sacro Cuore, protocol ID: 5117 (no-profit study), 03-08-2022. A written informed consent was obtained from all the involved subjects. The studies were conducted in accordance with the local legislation and institutional requirements. The participants provided their written informed consent to participate in this study. Written informed consent was obtained from the individual(s) for the publication of any potentially identifiable images or data included in this article.

## Author contributions

MD: Formal analysis, Investigation, Writing – original draft, Writing – review & editing. OG: Formal analysis, Investigation, Writing – original draft, Writing – review & editing. FS: Formal analysis, Investigation, Writing – original draft, Writing – review & editing. FM: Formal analysis, Writing – review & editing. GT: Conceptualization, Supervision, Writing – review & editing. RS: Conceptualization, Funding acquisition, Supervision, Writing – review & editing. AM: Conceptualization, Supervision, Writing – review & editing. LT: Investigation, Writing – review & editing. AF: Conceptualization, Funding acquisition, Project administration, Writing – review & editing. MS: Supervision, Writing – original draft, Writing – review & editing.
