# Supplementary material for: NMR-based metabolomics for investigating urinary profiles of metal carpentry workers exposed to welding fumes and volatile organic compounds
Source: Front Public Health. 2024 Aug 7;12:1386441. doi: 10.3389/fpubh.2024.1386441 (PMC11335539; doi:10.3389/fpubh.2024.1386441)
Supplement: Supplementary file 1 [file Data_Sheet_1.docx]

Supplementary Material

# Supplementary Figures and Tables

## Supplementary Figures


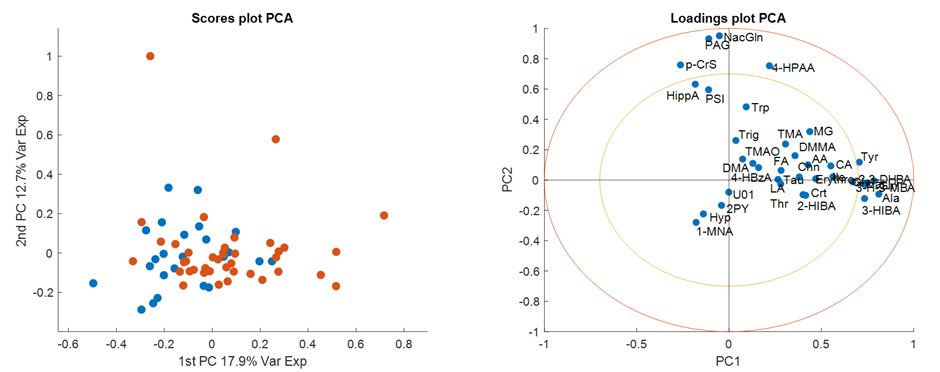


**Supplementary Figure 1.** PCA performed on CTRL (blue) and welding fumes exposed workers (orange). A) scores plot, B) loadings plot.


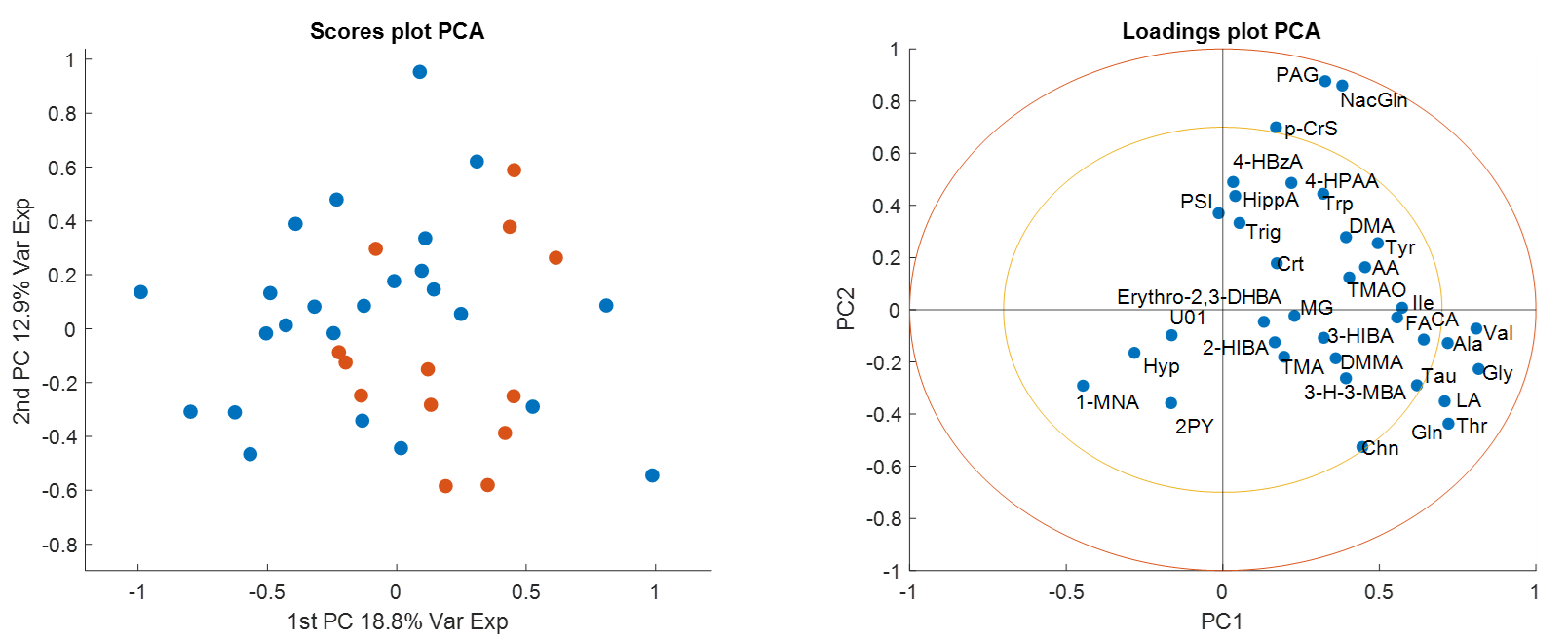


**Supplementary Figure 2**. PCA performed on CTRL (blue) and VOC exposed workers (orange). A) scores plot, B) loadings plot.


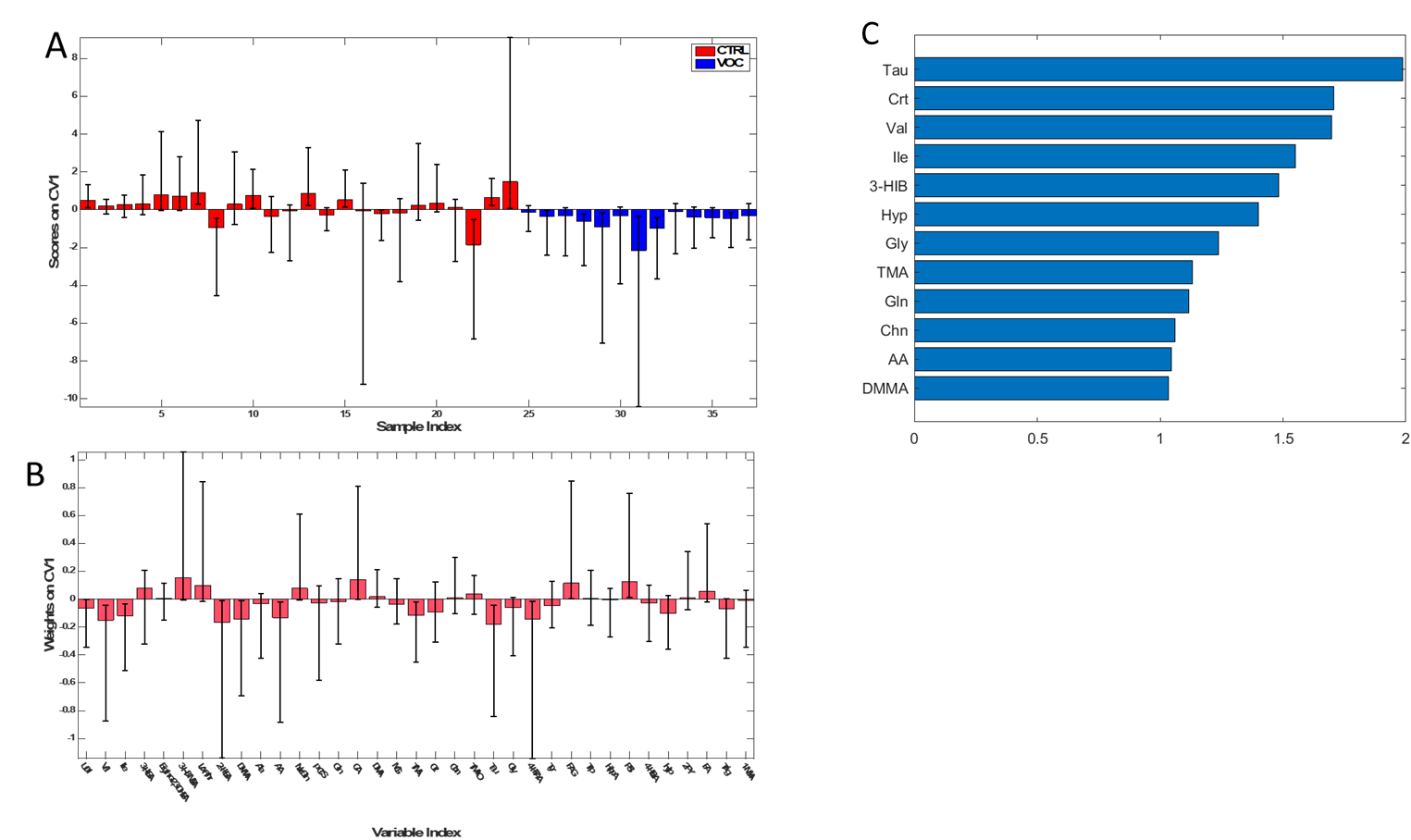


**Supplementary Figure 3**. PLS-DA model for classifying VOC and Ctrl. A) sample scores; B) variables weights along the only direction of maximum discrimination (first canonical variate) of the model; C) VIP scores of discriminant metabolites.

## Supplementary Tables

Supplementary table 1. ^1^H and ^13^C resonance assignments; s: singlet, d: doublet, t:triplet, q: quartet, dd: doublet of doublets, m: multiplet, bs: broad singlet. In bold the resonances considered for the quantification are reported.

| **Molecule** | **Structure** | **^1^H δ (ppm)** | **Assignment** | **Multiplicity** | **^13^C δ (ppm)** |
| --- | --- | --- | --- | --- | --- |
| 1. U01 |  | **0.75**  0.88  2.16 |  | **d** |  |
| 1. Valine (Val) | 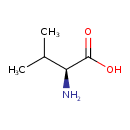 | **0.99**  1.04  ?  ? | **CH3**  CH3  β-CH  α-CH | **d**  d  m  d |  |
| 1. Isoleucine (Ile) | 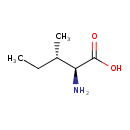 | **1.01** | **β-CH3** | **d** |  |
| 1. 3-Hydroxyisobutyric acid (3-HIBA) | 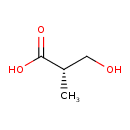 | **1.08**  2.49  3.53  3.70 | **CH3**  α-CH  β-CH  β-CH’ | **d** | 16.00 |
| 1. Erythro-2,3- dihydroxybutyric acid (Erythro-2,3-DHBA) | 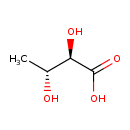 | **1.11**  4.12 | **CH3**  α-CH | **d** |  |
| 1. 3-Hydroxy-3-methylbutyric acid (3-H-3-MBA) | 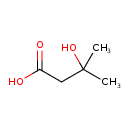 | **1.27**  2.37 | **CH3, CH3’**  CH2 | **s**  s | 30.90 |
| 1. Lactic acid (LA) | 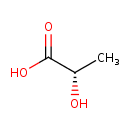 | **1.33**  4.11 | **CH3**  α-CH | **d**  q | 22.90 |
| 1. Threonine (Thr) | 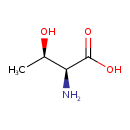 | **1.34**  3.60  4.27 | **CH3**  α-CH  β-CH | **d** | 22.90 |
| 1. 2-Hydroxyisobutyric acid (2-HIBA) | 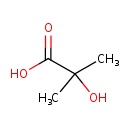 | **1.36** | **CH3, CH3’** | **s** | 29.5 |
| 1. Dimethylmalonic acid (DMMA) | 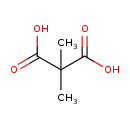 | **1.44** | **CH3, CH3’** | **s** |  |
| 1. Alanine (Ala) | 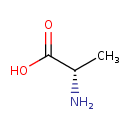 | **1.48**  3.78 | **CH3**  α-CH | **d**  q | 19.15 |
| 1. Acetic acid (AA) | 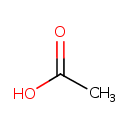 | **1.93** | **CH3** | **s** | 26.30 |
| 1. N-acetylglutamine (NAcGln) | 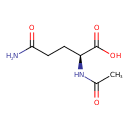 | 1.94  2.16  **2.27**  4.19  7.97 | β-CH  β’-CH  **γ-CH2**  α-CH  NH | m  m  **m**  m  bd | 34.7 |
| 1. Glutamine (Gln) | 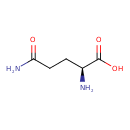 | 2.13  **2.46**  3.78 | β -CH2  **γ-CH2**  α-CH | m  **m**  dd | 33.83 |
| 1. p-Cresol sulfate (p-CrS) | 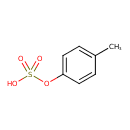 | **2.35**  7.20  7.29 | **CH3**  2,4-CH  3,5-CH | s | 22.5 |
| 1. Citric acid (CA) | 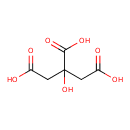 | **2.54**  2.69 | **α,β-CH2**  α’,β’-CH2 | **d**  d | 48.0  48.0 |
| 1. Dimethylamine (DMA) | 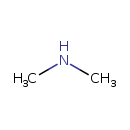 | **2.73** | **CH3, CH3’** | **s** | 37.9 |
| 1. Methylguanidine (MG) | 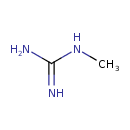 | **2.83** | **CH3** | **bs** | 30.10 |
| 1. Trimethylamine (TMA) | 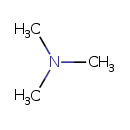 | **2.89** | **CH3, CH3’, CH3’’** | **s** | 45.80 |
| 1. Creatine (Crt) | 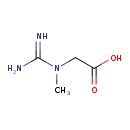 | 3.05  **3.94** | CH3  **CH2** | **s**  s | 39.80  56.85 |
| 1. Creatinine (Crtn) | 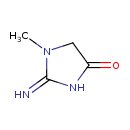 | 3.05  **4.06** | CH3  **CH2** | s  **s** | 32.96  59.32 |
| 1. Choline (Chn) | 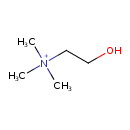 | **3.22** | **N-CH3,CH3’,CH3’’** | **s** | 57.20 |
| 1. Taurine (Tau) | 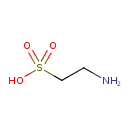 | 3.27  **3.43** | CH2  **CH2** | t  **t** | 50.50  38.66 |
| 1. Trimethylamine-N-Oxide (TMAO) | 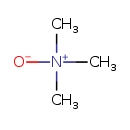 | **3.27** | **CH3, CH3’, CH3’’** | **s** | 62.08 |
| 1. Glycine (Gly) | 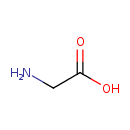 | **3.57** | **CH2** | **s** | 44.5 |
| 1. 4-Hydroxyphenylacetic acid (4-HPAA) | 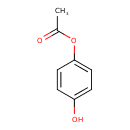 | **6.86**  7.16 | **3,5-CH**  2,6-CH | **dd**  dd |  |
| 1. Tyrosine (Tyr) | 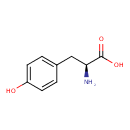 | **6.90**  7.19 | **3,5-CH**  2,6-CH | **dd**  dd |  |
| 1. 4-Hydroxybenzoic acid (4-HBzA) | 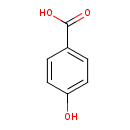 | 6.97  **7.76** | 3,5-CH  **2,6-CH** | d  **d** | 132.18 |
| 1. Tryptophan (Trp) | 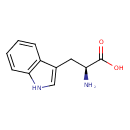 | 7.20  7.27?  7.29?  **7.50**  7.70 | 3-CH  2-CH  **5-CH**  4-CH | **pd**  pd | 115.23 |
| 1. Phenylacetylglycine (PAG) | 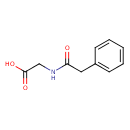 | **7.34-7.43** | **2-5 CH** | **m** | 131.5 |
| 1. Hippuric acid (HippA) | 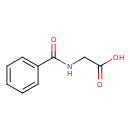 | 3.97  **7.55**  7.64  7.83 | CH2  **3,5-CH**  4-CH  2,6-CH | d  **m**  m  m | 46.4  131.71  135.18  130.09 |
| 1. Pseudouridine (PSI) | 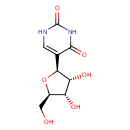 | **7.67** | **CH** | **s** | 144.5 |
| 1. Hypoxanthine (Hyp) | 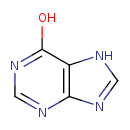 | **8.19**  8.21 | **2-CH**  7-CH | **s**  s |  |
| 1. N1-Methyl-2-pyridone-5-carboxamide (2PY) | 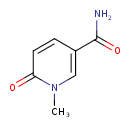 | 3.64  6.67?  7.98?  **8.33** | N-CH3  3-CH  4-CH  **6-CH** | s  d  d  **dd** | 182.12 |
| 1. Formic acid (FA) | 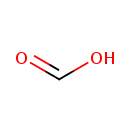 | **8.46** | **CH** | **s** | 169.23 |
| 1. Trigonelline (Trig) | 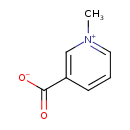 | 4.34  8.08  8.84  **9.12** | N-CH3  5-CH  4,6-CH  **2-CH** | s  m  m  **s** | 148.5 |
| 1. 1-Methylnicotinamide (1-MNA) | 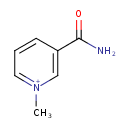 | 4.44  8.17  8.89  8.96  **9.28** | N-CH3  5-CH  4-CH  6-CH  **2-CH** | s  t  d  d  **s** |  |
